# Mendelian randomization and transcriptome analysis identified immune-related biomarkers for osteoarthritis

**DOI:** 10.3389/fimmu.2024.1334479

**Published:** 2024-04-12

**Authors:** Wei-Wei Pang, Yi-Sheng Cai, Chong Cao, Fu-Rong Zhang, Qin Zeng, Dan-Yang Liu, Ning Wang, Xiao-Chao Qu, Xiang-Ding Chen, Hong-Wen Deng, Li-Jun Tan

**Affiliations:** ^1^ Laboratory of Molecular and Statistical Genetics, College of Life Sciences, Hunan Normal University, Changsha, Hunan, China; ^2^ Tulane Center of Biomedical Informatics and Genomics, Deming Department of Medicine, School of Medicine, Tulane University, New Orleans, LA, United States

**Keywords:** osteoarthritis, mendelian randomization, multi-omics data, immune microenvironment, biomarkers

## Abstract

**Background:**

The immune microenvironment assumes a significant role in the pathogenesis of osteoarthritis (OA). However, the current biomarkers for the diagnosis and treatment of OA are not satisfactory. Our study aims to identify new OA immune-related biomarkers to direct the prevention and treatment of OA using multi-omics data.

**Methods:**

The discovery dataset integrated the GSE89408 and GSE143514 datasets to identify biomarkers that were significantly associated with the OA immune microenvironment through multiple machine learning methods and weighted gene co-expression network analysis (WGCNA). The identified signature genes were confirmed using two independent validation datasets. We also performed a two-sample mendelian randomization (MR) study to generate causal relationships between biomarkers and OA using OA genome-wide association study (GWAS) summary data (cases n = 24,955, controls n = 378,169). Inverse-variance weighting (IVW) method was used as the main method of causal estimates. Sensitivity analyses were performed to assess the robustness and reliability of the IVW results.

**Results:**

Three signature genes (FCER1G, HLA-DMB, and HHLA-DPA1) associated with the OA immune microenvironment were identified as having good diagnostic performances, which can be used as biomarkers. MR results showed increased levels of FCER1G (OR = 1.118, 95% CI 1.031-1.212, P = 0.041), HLA-DMB (OR = 1.057, 95% CI 1.045 -1.069, P = 1.11E-21) and HLA-DPA1 (OR = 1.030, 95% CI 1.005-1.056, P = 0.017) were causally and positively associated with the risk of developing OA.

**Conclusion:**

The present study identified the 3 potential immune-related biomarkers for OA, providing new perspectives for the prevention and treatment of OA. The MR study provides genetic support for the causal effects of the 3 biomarkers with OA and may provide new insights into the molecular mechanisms leading to the development of OA.

## 1 Introduction

Osteoarthritis (OA) is a common and disabling chronic degenerative joint disease with major pathological changes including articular cartilage degeneration, bone fragmentation, mild synovial inflammation, and subchondral bone remodeling (1, 2). With the continuing global obesity epidemic and an aging population, the prevalence of OA is gradually increasing, resulting in a substantial medical and economic burden (3). The etiology of OA has conventionally been attributed to mechanical strain causing the deterioration of cartilage, thereby characterizing it as a non-inflammatory condition. However, recent studies have shown that OA is inflammatory, accompanied by multiple immune cells infiltrating the synovial membrane (4, 5). Moreover, the synovial immune microenvironment in OA promotes cartilage injury or repair (6). At present, pharmacological treatments for OA are mostly limited to pain relief rather than immunotherapy based on restoring damage to joint structures and reducing inflammation (7). Therefore, it is necessary to identify reliable biomarkers associated with the OA immune microenvironment and guide the personalized treatment of OA patients.

Studies have increasingly demonstrated that changes in immune cells in the synovium play a pivotal role in synovial inflammation and cartilage damage and repair (8, 9). For example, M1 macrophages within the synovium exacerbate synovitis and cartilage degeneration by secreting pro-inflammatory factors (IL1β) and matrix metalloproteinase (MMPs) (10). In contrast, M2 macrophages express anti-inflammatory factors (IL10) and growth factors (TGF-β) that accelerate the regression of inflammation and contribute to cartilage repair (11, 12). Type 1 T helper (Th1) cells and type 17 T helper (Th17) cells are involved in OA progression by releasing inflammatory cytokines (13, 14). Inflammatory and immunostimulatory cytokines released by mature dendritic cells (DCs) aggravate synovial inflammation and cartilage degradation (15). In contrast, immature DCs promote the proliferation of regulatory T cells and induce cartilage differentiation in mesenchymal stem cells (MSCs), thus aiding in cartilage repair (16). Together, these findings emphasized the critical role of the immune cells in OA. Consequently, considering the potential of immunotherapy to alleviate the symptoms of OA, it is crucial to meticulously explore the signature genes associated with the OA immune microenvironment.

Mendelian randomization (MR) is a method used to infer causal associations between exposures and outcomes (17). The MR approach has been widely used to identify molecular markers that contribute to the development of diseases, which means that causal relationships between genes and diseases can be inferred by using expression quantitative trait loci (eQTL) variants of genes as instrumental variables (18, 19). Previous studies have examined the OA immune microenvironment and immune-related genes only through transcriptomic data (20, 21). In this study, we comprehensively explored the OA synovial immune microenvironment patterns and explored immune-related biomarkers of OA using transcriptomics and genomics data. Single-sample gene set enrichment analysis (ssGSEA), machine learning, and weighted gene co-expression network analysis (WGCNA) were used to explore the signature genes associated with the OA immune microenvironment as potential new biomarkers. Moreover, we investigated the expressions of signature genes at single-cell resolution and cellular communication. We inferred the causal relationships between biomarkers and OA through a MR study. Overall, we used multi-omics data to explore signature genes associated with the OA immune microenvironment as novel biomarkers, providing resources for the accurate diagnosis and treatment of OA.

## 2 Materials and methods

### 2.1 Data acquisition and processing

**Figure 1:**
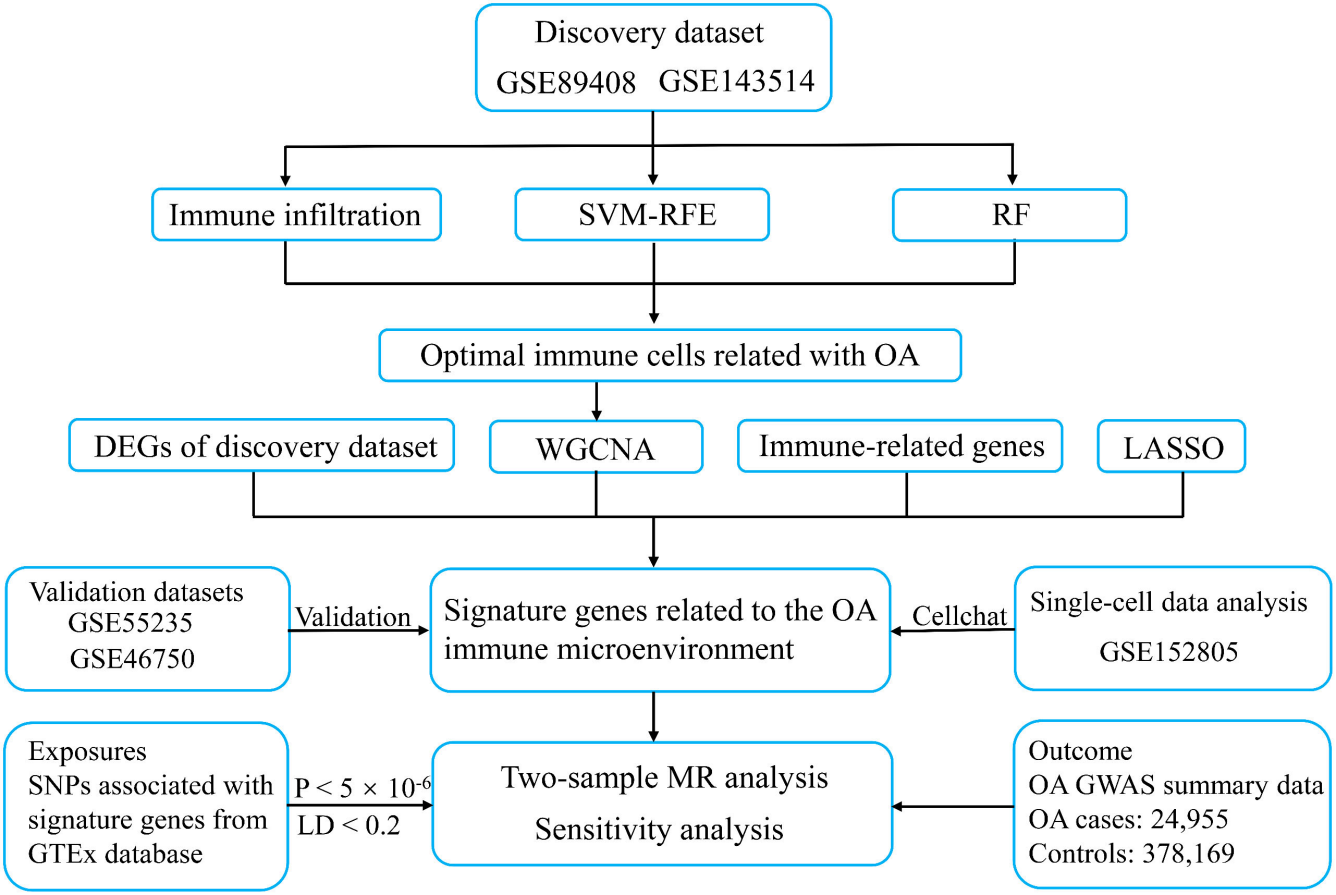
The workflow of the present study. SVM-RFE, support vector machine recursive feature elimination; RF, random forest; WGCNA, weighted gene co-expression network analysis; LASSO, least absolute shrinkage and selection operator; OA, osteoarthritis; MR, mendelian randomization; LD, linked disequilibrium.

We retrieved publicly available five transcriptome datasets (GSE89408, GSE143514, GSE55235, GSE46750, and GSE152805) from Gene Expression Omnibus (GEO) database using the keywords “osteoarthritis”, “synovium”, and “Homo sapiens” (22–26). The inclusion criteria were as follows: Firstly, the datasets were derived from synovial tissue of human knee OA. Secondly, only datasets from published articles were considered to ensure data quality. Finally, datasets containing at least 10 OA and 10 healthy samples were used as the validation datasets to make the validation results more convincing. The details of the datasets included in this study are shown in **Supplementary Table 1**. Two transcriptome sequencing datasets (GSE89408 and GSE143514) were integrated into a discovery dataset containing 31 normal tissues and 27 OA synovial tissues after applying the R package “sva” to remove batch effects. The R package “DEseq2” was used to calculate differentially expressed genes (DEGs) from the discovery dataset, taking adjusted p-value < 0.05 and |log2 FC| ≥ 1 as the criteria for statistical significance. Two normalized microarray datasets (GSE55235 and GSE46750) were utilized as the validation sets. The single-cell dataset (GSE152805) was analyzed after quality control, normalization, downscaling, and cell annotation using cellular marker genes. The study design is shown in **Figure 1**.

### 2.2 Evaluation of immune infiltration

The immune score and immune infiltration analyses were conducted using the single-sample gene set enrichment analysis (ssGSEA) method in the “GSVA” R package. Immune score analysis was an overall assessment of the abundance of immune cells in the OA synovium. Immune infiltration analysis was performed to assess the expression of marker genes for 28 immune cell types in the immune cell gene set, aiming to infer the abundance of each immune cell in the OA synovium from the discovery dataset. The ssGSEA is an extension and improvement of the gene set enrichment analysis (GSEA) method (27), which defines enrichment scores based on the ranking of gene expression levels and is used to assess the degree of enrichment of specific gene sets in each sample. ssGSEA score provided a way to quantify the relative abundance of immune cells in OA synovial tissues and was able to assess the level of immune infiltration in each sample. Wilcoxon test was applied to assess the difference in the abundance of immune cells between the normal and OA groups, with p-values less than 0.05 being considered statistically significant.

### 2.3 Identification of characteristic immune cells

Support vector machine recursive feature elimination (SVM-RFE) and random forest (RF) algorithms were used to determine the optimal immune cells related with OA (28, 29). We used the two methods to ensure that the shared results were more robust and reliable. SVM-RFE and RF analyses were performed by the “e1071” and “randomForest” R packages, respectively. SVM-RFE is a support vector machine approach based on recursive feature elimination (RFE) to find the best variables. The RF algorithm constructs multiple decision trees through the sampling of objects and variables. Sequentially, the objects are classified, and the relative importance of the variables to the classification is measured while the classification is being performed. We selected immune cell types with mean Gini importance > 2 as the characteristic immune cells in the RF analysis. Finally, the shared immune cells in the results of two algorithms were identified as the characteristic immune cells.

### 2.4 Construction and validation of signature genes related to the OA immune microenvironment

Utilizing the “WGCNA” R package, weighted gene co-expression network analysis (WGCNA) was used to find modules of highly related genes and explore the relationships between modules and specific traits (30). In our study, we used characteristic immune cells as traits and explored co-expressed gene modules that had the highest significant positive correlation with characterized immune cells. The optimal soft-threshold power was selected as the first power value that reached a scale-free topology index of 0.85. Immune-related genes were obtained from ImmPort (https://www.immport.org/home) database (31). The DEGs associated with the OA immune microenvironment were identified using the intersection of immune-related genes, DEGs, and genes from modules significantly correlated with all characteristic immune cells. We further explored the biological pathways and functions involved in the DEGs through the Metascape (http://metascape.org) database (32). Based on the DEGs, we employed the “e1071” R package to execute the least absolute shrinkage and selection operator (LASSO) algorithm for the selection of signature genes (33). LASSO regression typically generates a penalty function to filter the variables to prevent overfitting the model when there are too many variables and the sample size of the dataset is small. The signature genes filtered by the LASSO algorithm were validated and evaluated for diagnostic confidence in OA by two independent validation datasets (GSE55235 and GSE46750). Finally, the validated signature genes were considered as immune related biomarkers for OA, and the R package “pROC” was applied to plot the AUC curves of the biomarkers to visualize the diagnostic effect.

### 2.5 Gene set enrichment analysis

We conducted Gene Set Enrichment Analysis (GSEA) to further explore the involved prospective molecular mechanisms for the signature genes. The pathway gene sets were obtained from the Molecular Signature Database (MSigDB) (https://www.gsea-msigdb.org/gsea/msigdb/) (34). Based on the gene expression profile data of the discovery dataset, the median expression values of signature genes were calculated separately. All samples in the dataset were divided into high and low expression groups based on whether the expression value of the signature gene was higher or lower than its median. The GSEA method used the differentially expressed gene list between the two groups and predefined pathway gene sets to explore significantly enriched pathways. Through GSEA analysis based on the grouping of signature gene expression levels, we can better understand the biological pathways associated with different signature gene expression levels in OA. Enrichment results were considered significant when they met the following statistical thresholds: p-value < 0.05, |normalized enrichment score (NES)| > 1, and false discovery rate (FDR) < 0.25 (35).

### 2.6 Correlation of signature genes with OA related disease genes and inflammatory genes

To explore potential correlations between signature genes and OA related disease genes, we obtained OA genes of the knee from the DisGeNet database (36). The DisGeNET database (http://www.disgenet.org/) is a database of disease-associated genes integrating data from expert curated repositories, GWAS catalogues, animal models, and the scientific literature. We obtained a set of inflammation-associated genes (GOBP_INFLAMMATORY_RESPONSE) from the MSigDB and performed differential expression analysis between the normal and OA groups in the discovery dataset. Based on the gene expression values, the Hmisc package was used to investigate the correlations between inflammatory genes differentially expressed in OA and signature genes. Finally, to uncover the correlation between signature genes and immune cells in the OA immune microenvironment, we also performed a correlation analysis between signature genes and immune cells based on gene expression values and enrichment scores of immune infiltration analysis.

### 2.7 Expression of signature genes and cellular communication in single-cell data

To obtain a more comprehensive understanding of the roles of signature genes correlated with the immune microenvironment in the progression of OA, we analyzed the single-cell RNA (scRNA) sequencing data containing 3 OA synovial samples. The single-cell expression matrix data from GSE152805 were downloaded, and data processing was performed using the Seurat package, including filtering of low-quality cells, data normalization, variable feature identification, data scaling, and principal component analysis. The three samples were integrated with CCA (cross-dataset normalization) algorithm. Cells were clustered at the appropriate resolution utilizing the “FindClusters” function and then visualized using the “RunUMAP” function. Cellular annotation of processed single-cell data was based on marker genes provided by the authors of the data (26). We assessed the expression of signature genes in different immune cell clusters. Cell-cell communication analyzes the expression of ligand-receptor pairs in different cell types and reveals specific signaling pathways between cell types. The immune cell clusters in which signature genes were involved in cell-cell communication were elucidated by CellChat (37).

### 2.8 MR analysis of biomarkers and OA

Two-sample MR analysis was performed to assess the causality between biomarkers and OA using the TwoSample package (38). The expressions of biomarkers were used as exposure factors and OA as an outcome. Since cis-eQTLs have a more direct regulatory effect on gene expression, this study used SNPs from cis-eQTLs of biomarkers in the GTEx database (https://www.gtexportal.org/) as instrumental variables (IVs) (39). SNPs (physical distance threshold 10,000 kb, linkage disequilibrium threshold of r^2^ < 0.2) at genome-wide significance (P < 5 × 10^-6^) were included in the MR analysis. The GWAS summary data for OA were extracted from a GWAS meta-analysis based on the UK Biobank and the Arthritis Research UK Osteoarthritis Genetics (arcOGEN) resource, which included 24,955 knee osteoarthritis cases and 378,169 controls (40). To explore the causal effects of biomarkers expression on OA, three MR analysis methods were used in this study. We applied the random-effects inverse-variance weighting (IVW), the weighted median (WM), and the MR-Egger method making different assumptions about horizontal multiplicity were used as complements to the IVW to test the robustness of the MR analysis. Estimates from the random-effects IVW method were used as the primary results because this method is the most efficient method for MR analysis (41). The WM method is robust in the presence of outliers and can provide a consistent estimate of the causal effect even if 50% of the IVs are invalid (42). MR-Egger regression can provide a test of horizontal pleiotropy, but its estimates generally exhibit low precision (43).

### 2.9 Sensitivity analyses

Sensitivity analyses, including tests for horizontal pleiotropy and heterogeneity, were also performed to validate the MR hypothesis and to ensure the robustness of the causal associations of the identified candidate biomarkers. We evaluated the heterogeneity between the causal estimates of each SNP using the Cochran’s Q test. Specifically, the P value of Cochran’s Q test less than 0.05 was considered to be significantly heterogeneous. Even in the presence of heterogeneity, the random-effects IVW test also provided reliable causal estimates in the absence of horizontal pleiotropy (41). The leave-one-out analysis was conducted to assess whether any individual SNP would cause bias in the MR results. The horizontal pleiotropy between SNPs was detected by the MR-Egger intercept test (44). Steiger filtering was also performed to determine the directionality of the relationship between IVs of biomarkers and OA (45). When the Bonferroni-corrected p-value threshold was less than 0.017 (correcting for 3 exposures and 1 outcome), the correlation was considered to have statistical significance, while a p-value less than 0.05 was regarded as nominally significant evidence of a potential causal association (46).

## 3 Results

### 3.1 Assessment of immune infiltration in OA patients

**Figure 2:**
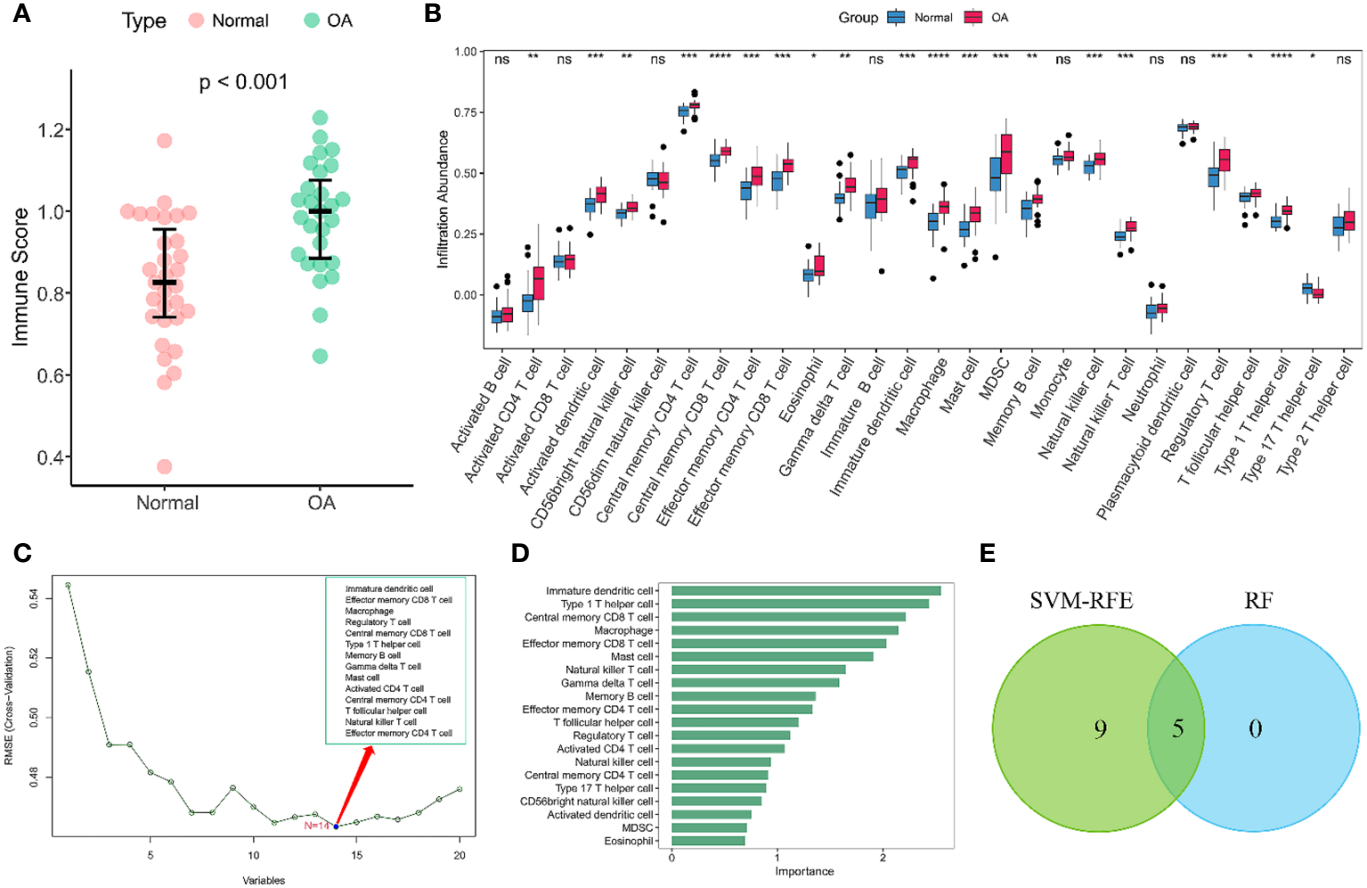
Immune infiltration analysis of OA. **(A)** Scatterplot displaying the differences in immune scores between OA patients and normal individuals. Normal (n = 31), OA (n = 27). **(B)** Boxplots of the immune infiltration abundance in OA and non-OA individuals. Normal (n = 31), OA (n = 27). **(C)** The SVF-RFE algorithm screens out 14 immune cells. **(D)** Ranking of immune cells based on importance scores by RF algorithm. **(E)** The Venn diagram shows that five characteristic immune cell types are determined by the two algorithms described above. Statistical comparisons obtained by the Wilcoxon test in **(A, B)** (ns not significant, p > 0.05, *p < 0.05, **p < 0.01, ***p < 0.001, ****p < 0.0001).

Given the importance of changes in the immune microenvironment contributing to the progression of OA, we compared the immune scores of OA and normal individuals in the discovery dataset, which provided a qualitative assessment of the immune microenvironment (**Figure 2A**). OA patients had higher immune scores than normal individuals, demonstrating that immune cells were more abundant in OA. Next, we specifically examined the differences in the enrichment scores of 28 immune cell types in the OA and healthy groups. The results showed that 20 immune cells had significantly different immune infiltration levels between the normal and OA groups (**Figure 2B**). Compared to controls, OA patients had higher levels of T cell infiltration, including central memory CD4 T cells, central memory CD8 T cells, regulatory T cells, and Th1 cells. Moreover, macrophages, myeloid-derived suppressor cells (MDSC), immature dendritic cells, mast cells also had high level of infiltration. To further identify the characteristic immune cells associated with OA progression, we performed SVM-RFE and RF analyses. The results of the SVM-RFE algorithm selected 14 immune cells (**Figure 2C**), while the RF analysis showed five different immune cells with an importance of more than 2 (**Figure 2D**). Finally, based on the shared immune cells of both algorithms, we identified five immune cells as characteristic immune cells, namely central memory CD8 T cells, effector memory CD8 T cells, immature dendritic cells, macrophages, and Th1 cells (**Figure 2E**).

### 3.2 Identification of immune-related signature genes

**Figure 3:**
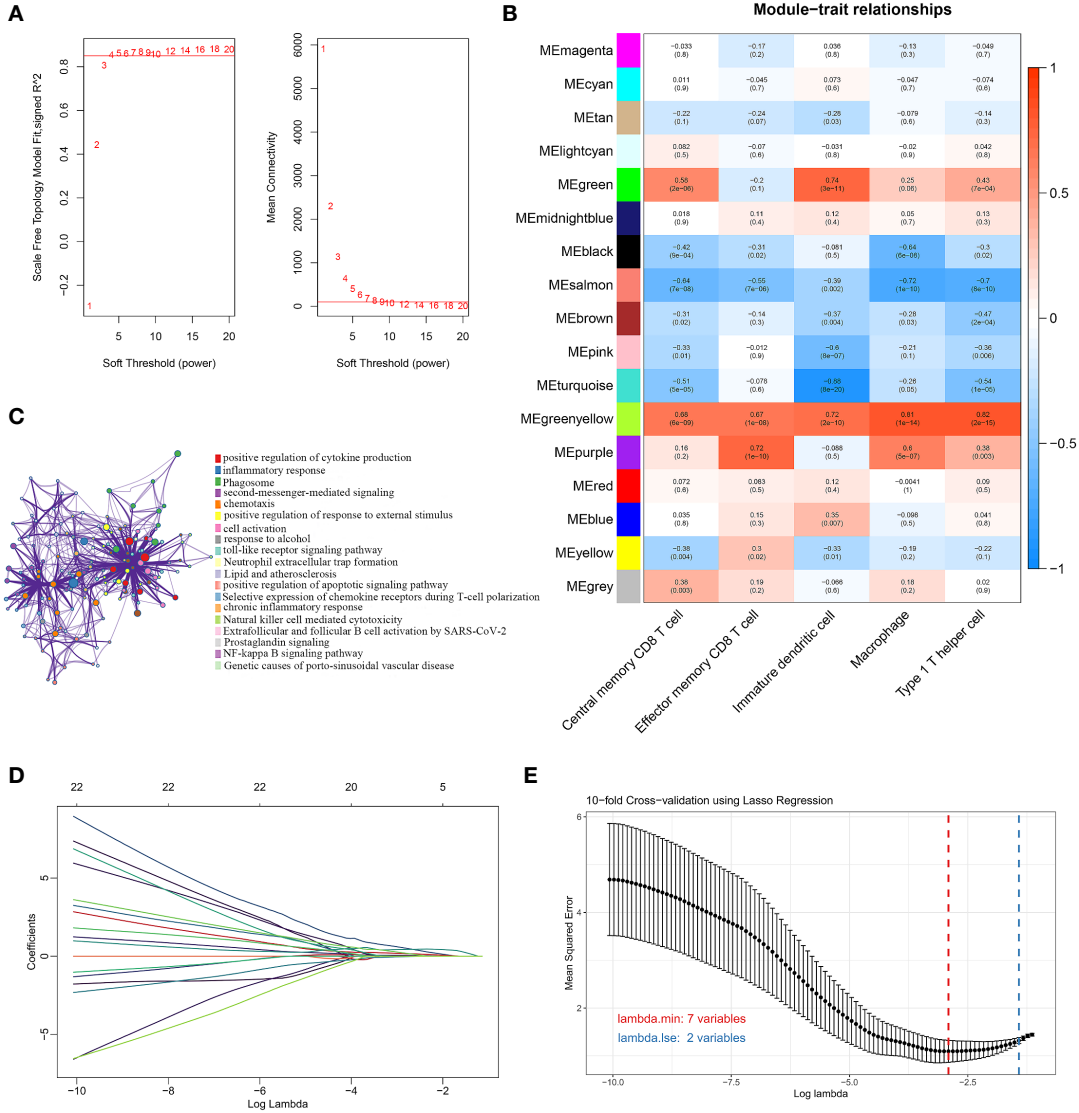
Screening for immune-related signature genes. **(A)** Different soft threshold powers (β) and analysis of mean connectivity. **(B)** Heatmap of correlation between modules and five signature immune cells. **(C)** Enrichment analysis of GO and KEGG pathways related to 44 signature DEGs. **(D)** LASSO coefficient profiles of 44 DEGs. **(E)** Selection of the best parameter for nonzero coefficients by 10-fold cross-validation in the LASSO regression model.

We used the WGCNA algorithm to extract co-expressed gene modules significantly correlated with all five characterized immune cells. The power value of 4 was chosen as the optimal soft-thresholding power because it was the first power to reach a scale-free topology index of 0.85 (**Figure 3A**). The greenyellow module had a highly significant positive correlation with central memory CD8 T cells, effector memory CD8 T cells, immature dendritic cells, macrophages, and Th1 cells. In contrast, the salmon module had a significant negative correlation with the five characteristic immune cells (**Figure 3B**). The greenyellow module had the strongest positive correlation with central memory CD8 T cells, macrophages, and Th1 cells, respectively. The purple module showed the strongest positive correlation with effector memory CD8 T cells, while immature dendritic cells had the highest positive correlation with the green module. Therefore, the genes in the greenyellow module, the purple module, and the green module were selected for the following analysis. Using the genes in the three modules, DEGs between OA and normal samples, and immune-related genes from the ImmPort database, we identified 44 DEGs associated with the immune microenvironment (**Supplementary Table 2**). The biological pathways and functions involved in the 44 immune microenvironment-related DEGs were mainly enriched in positive regulation of cytokine production, inflammatory response, positive regulation of response to external stimulus, toll-like receptor signaling pathway, and NF-kappa B signaling pathway (**Figure 3C**), suggesting that the immune microenvironment-related DEGs were implicated in the pathogenesis of OA. To further filter for signature genes associated with the OA immune microenvironment, the 7 signature genes (HLA-DPA1, SEMA3A, FCER1G, HLA-DMB, INHBB, IL10, and OSM) were identified from 44 DEGs using the LASSO algorithm (**Figures 3D, E**). Compared to healthy controls, all 7 signature genes were upregulated in the OA of the discovery dataset.

### 3.3 Validation and evaluation of signature genes

**Figure 4:**
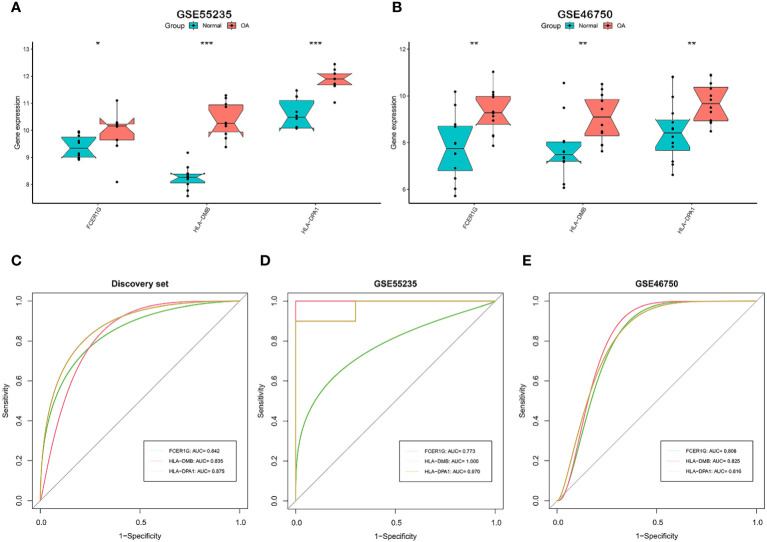
Validation and the diagnostic performance of immune-related signature genes. **(A, B)** Differential expression of the 3 signature genes in external independent validation datasets. GSE55235 **(A)** and GSE46750 **(B)**. **(C-E)** The receiver operating characteristic (ROC) curves and area under the curve (AUC) scores for the diagnostic performance of signature genes in the discovery dataset **(C)**, GSE55235 dataset **(D)**, and GSE46750 dataset **(E)**. GSE55235 (10 normal and 10 OA samples). GSE46750 (12 normal and 12 OA samples). The Wilcoxon test in **(A, B)** (*p < 0.05, **p < 0.01, ***p < 0.001).

We tested whether the 7 signature genes were also differentially expressed in two independent validation datasets, GSE55235 and GSE46750. Only the 3 signature genes, FCER1G, HLA-DMB, and HLA-DPA1, exhibited significant upregulated expression in both validation datasets. (**Figures 4A, B**). Therefore, these three signature genes associated with the OA immune microenvironment could serve as potential biomarkers for OA. Subsequently, the accuracy of the three signature genes for diagnosing OA was assessed using ROC curve analysis. All the AUC values of three signature genes, FCER1G, HLA-DMB, and HLA-DPA1, were greater than 0.7 in the discovery dataset and two independent validation datasets, suggesting that the 3 signature genes have good diagnostic performance as biomarkers of OA. (**Figures 4C–E**).

### 3.4 GSEA analysis of signature genes

**Figure 5:**
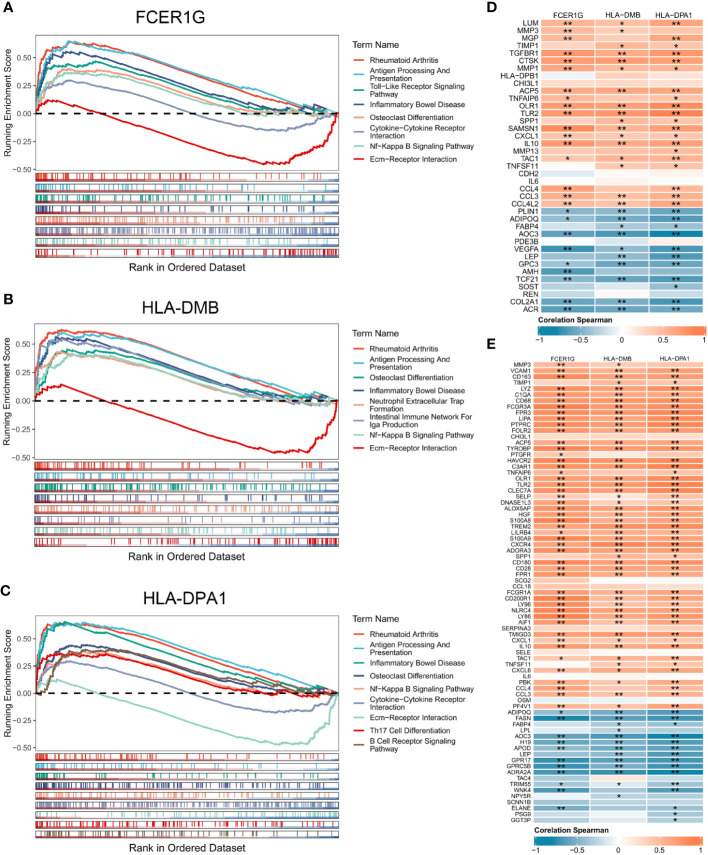
Gene set enrichment analysis of the 3 signature genes associated with the OA immune microenvironment. **(A-C)** GSEA displaying FCER1G **(A)**, HLA-DMB **(B)**, and HLA-DPA1 **(C)** enriched KEGG pathways, respectively. **(D)** Correlation analysis between the 3 signature genes and OA related genes. **(E)** Correlation analysis between the 3 signature genes and inflammatory genes. Normal (n = 31), OA (n = 27). Statistical comparisons obtained by the Wilcoxon test in B (*p < 0.05, **p < 0.01).

We performed GSEA to explore the biological pathways enriched by the 3 signature genes based on their expression values (**Figures 5A–C**). The 3 signature genes were mainly involved in rheumatoid arthritis, antigen processing and presentation, inflammatory bowel disease, ECM-receptor interaction, NF-Kappa B signaling pathway, and osteoclast differentiation. In addition, FCER1G was involved in cytokine-cytokine receptor interaction, while HLA-DPA1 participated in Th17 cell differentiation and B cell receptor signaling pathway. The above signaling pathways have been implicated in the immune response and inflammation, suggesting that the 3 signature genes are essential in the OA immune microenvironment and are involved in the inflammatory reaction in OA.

### 3.5 Correlation of signature genes with OA related genes, inflammatory genes and immune cells

To explore the contribution of signature genes in OA progression, we performed the correlation analysis of the 3 signature genes with OA related genes, inflammatory genes, and immune cells. OA genes obtained from the DisGeNET and DEGs shared 39 OA related genes that were upregulated or downregulated in OA synovium (**Supplementary Figure 1A**). The inflammatory genes were obtained from MSigDB sharing 77 DEGs (**Supplementary Figures 1B, C**). Correlation analysis of the 39 OA related genes and the 3 signature genes showed statistically significant correlations between the 3 signature genes and multiple OA related genes (**Figure 5D**). High expression of FCER1G was significantly positively correlated with the expression of IL10, SAMSN1, and CCL4. HLA-DM was notably correlated with CTSK and GPC3. HLA-DPA1 exhibited significant correlations with TLR2, COL2A1, and others. Furthermore, there were also high and significant correlations between the 3 signature genes and several inflammation-related genes (**Figure 5E**). Analysis of the correlation between signature genes and immune cells showed that the 3 signature genes were significantly associated with multiple immune cells in the OA immune microenvironment, such as macrophages, regulatory T cells, Th1 cells, and immature dendritic cells (**Supplementary Figures 2A-C**).

### 3.6 Single-cell analysis of signature genes associated with the OA immune microenvironment

**Figure 6:**
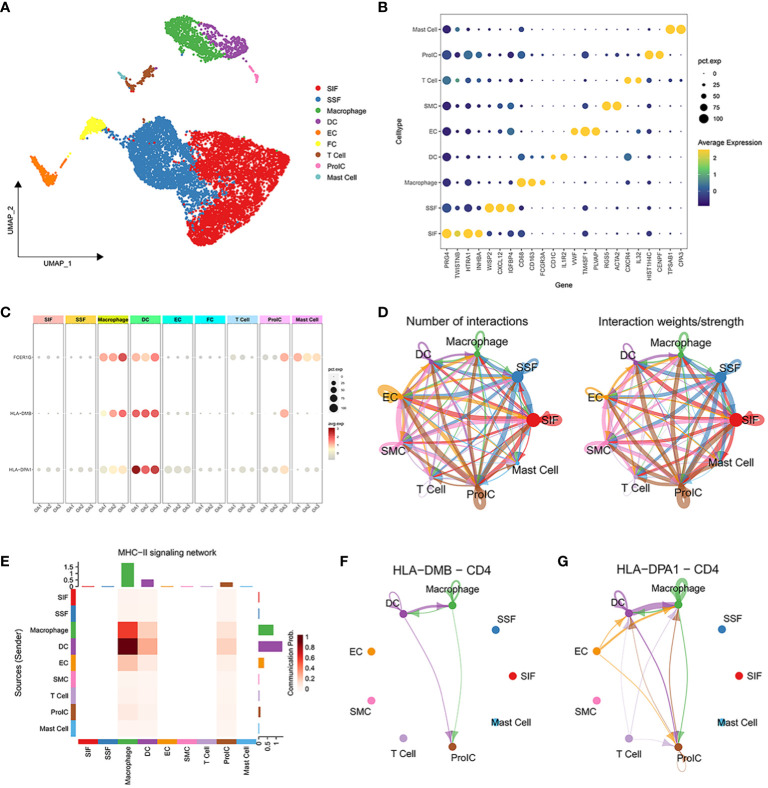
Single-cell transcriptome profiling of 3 OA synovial samples. **(A)** UMAP plot of all cells from 3 OA synovial samples. **(B)** The dot plot shows the average expression level (color scale) and percentage of cells expressing the marker genes (dot size) for each cluster. **(C)** Expression of characteristic genes in each cell cluster. **(D)** The number of interactions between 10 distinct cell types. **(E)** Heatmap of MHC-II signaling contributing predominantly to the sending or receiving of signaling of certain cell groups. **(F, G)** HLA-DMB ligand and HLA-DPA1 ligand for cell-cell communication with the CD4 receptor, respectively.

Based on the marker genes, we identified a total of 9 cell clusters: synovial subintimal fibroblasts (SSFs), synovial intimal fibroblasts (SIFs), macrophages, dendritic cells (DCs), endothelial cells (ECs), smooth muscle cells (SMCs), T cells, proliferating immune cells (ProICs), and mast cells, and visualized the marker genes of each cell cluster by dot plots (**Figures 6A, B**). we analyzed the expression of 3 signature genes associated with the immune microenvironment in each cell cluster (**Figure 6C**). The 3 signature genes were highly expressed in both macrophage clusters and dendritic cell clusters. To further explore the expression of signature genes in specific macrophage subtypes, we extracted the macrophage cluster and annotated two subpopulations of M1 and M2 macrophages (**Supplementary Figures 3A, B**). The 3 signature genes were all expressed in M1 macrophages (**Supplementary Figure 3C**), implying that M1 macrophages through the expression of signature genes may promote the advancement of inflammation in OA. Cell-cell interactions between 9 distinct cell types showed that significant interaction occurred amongst SSFs, SIFs, ECs, and immune cell clusters such as macrophages, DCs, and ProICs (**Figure 6D**). Cellchat analysis inferred that the HLA-DMB and HLA-DPA1 acted as ligands to mediate intercellular communication through the MHC-II signaling pathway. Through the analysis of the MHC-II signaling pathway between cells, it was observed that macrophages and DCs served as both senders and receivers in the OA immune microenvironment (**Figure 6E**). Notably, DCs primarily assumed the role of the main senders, while macrophages predominantly acted as the main receivers. Specifically, DCs expressed HLA-DMB and HLA-DPA1 and communicated with macrophages via the CD4 receptor (**Figures 6F, G**). In conclusion, the above results indicated that macrophages and DCs play crucial roles through the MHC-II signaling pathway in OA.

### 3.7 Causal effect of biomarkers on OA

**Figure 7:**
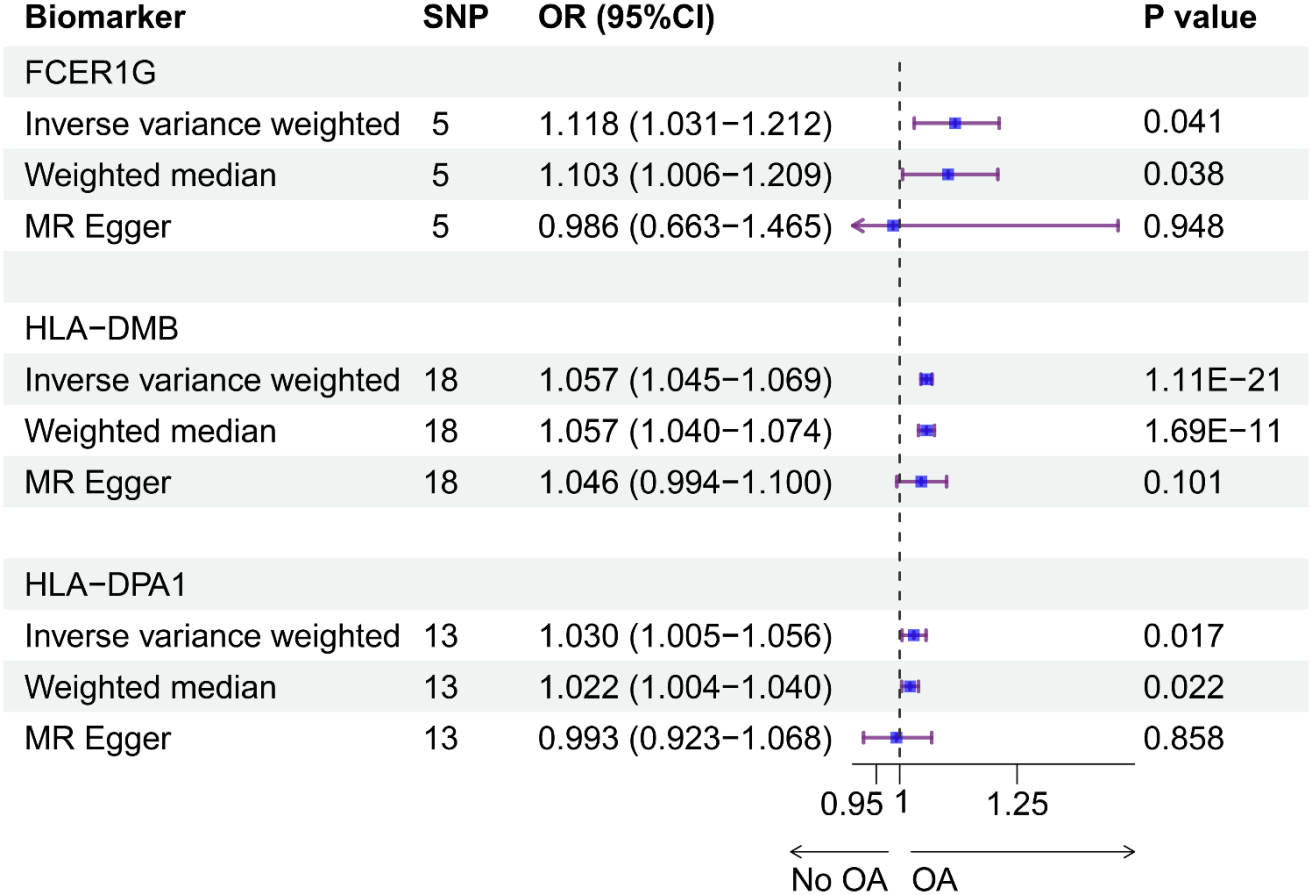
The causal effects between the biomarkers and OA were inferred by MR analysis. Forest plot showing causal estimates of biomarkers and risk of developing OA. The odds ratio (OR) was estimated using the IVW method. Horizontal bars represented 95% confidence intervals (CI).

Using the cis-eQTL instruments in IVW analysis, FCER1G (OR = 1.118, 95% CI 1.031-1.212, P = 0.041), HLA-DMB (OR = 1.057, 95% CI 1.045 -1.069, P = 1.11E-21) and HLA-DPA1 (OR = 1.030, 95% CI 1.005-1.056, P = 0.017) were all positively causally associated with OA (**Figure 7**). MR results of the WM method were consistent with those of IVW. Using MR-Egger analysis, we did not observe evidence of causal relationships between the 3 biomarkers and OA. To test the stability of the above results, the sensitivity analyses including Cochran’s Q test, MR-Egger intercept test were conducted (**Table 1**). The P values of MR-Egger intercept analysis for all 3 biomarkers were greater than 0.05, implying that there was no horizontal pleiotropy in the MR study. The P value of Cochran’s Q-test for HLA-DPA1 was less than 0.05, suggesting heterogeneity in MR studies of HLA-DPA1. However, MR-Egger intercept analysis did not detect any horizontal pleiotropy, suggesting that MR estimations did not introduce horizontal pleiotropy in the presence of heterogeneity of this biomarker. There was no heterogeneity in the results (P > 0.05) of Cochran’s Q test for HLA-DMB and FCER1G. Moreover, IVs with no potential effects were identified through the leave-one-out analysis (**Supplementary Figure S4**). Steiger filtering test ensured directional accuracy of the associations between biomarkers and OA (**Supplementary Table 3**). Thus, the increased expression levels of HLA-DPA1, HLA-DMB, and FCER1G are associated with an increased risk of OA.

**Table 1:** Heterogeneity (Cochran’s Q test) and horizontal pleiotropy (MR-Egger intercept test) tests for causal relationships between biomarkers and OA.

Exposure	Outcome	Cochran’s Q test		MR-Egger	
		Q value	P	Intercept	P
FCER1G	OA	1.212	0.876	0.018	0.570
HLA-DMB	OA	12.519	0.768	0.005	0.683
HLA-DPA1	OA	54.700	< 0.05	0.023	0.319

## 4 Discussion

Due to the aging and obesity epidemic, the prevalence of OA has increased dramatically, producing a significant impact on health and quality of life (3). Recent studies have shown that the OA immune microenvironment is closely related to synovial inflammation, cartilage damage, and repair (47). However, there is a lack of comprehensive studies on the immune microenvironment in OA. Consequently, this study systematically explored immune-related biomarkers for OA by bioinformatics approaches using genomics and transcriptomics data.

In this study, we identified five key immune cell types including central memory CD8 T cells, effector memory CD8 T cells, immature dendritic cells, macrophages, and Th1 cells which were significantly increased in OA. Furthermore, our study identified 3 signature genes (FCER1G, HLA-DMB, and HLA-DPA1) associated with the OA immune microenvironment, which can be used as immune-related biomarkers for OA. FCER1G, a component of the high-affinity immunoglobulin E (IgE) receptor, may be involved in IgE-mediated mast cell activation promoting synovitis and cartilage destruction in OA following mechanical injury in mice (48). Moreover, the IgE antibody was found to effectively bloc IgE-induced M1 macrophage polarization activity and reverse IgE-decreased M2 macrophage polarization, revealing that IgE regulated macrophage polarization towards a pro-inflammatory M1 phenotype (49). Compared with healthy controls, the expression of FCER1G was increased in cartilage from OA patients (50). HLA-DMB and HLA-DPA1, as major histocompatibility complex class II (MHC II) genes, may be involved in the regulation of OA by presenting antigens through MHC II molecules. Type II collagen-specific T regulatory cells were activated upon interacting with Col II (type II collagen) presented on MHC II of antigen-presenting cells (APCs), such as macrophages and DCs, at the OA synovium (51, 52). Cytokines secreted by activated T regulatory cells could inhibit Th1 cells and M1 macrophages in OA joints, thus reducing local inflammation (53). The Th1 cells played a role in OA synovial inflammation and cartilage degeneration by secreting inflammatory cytokines (8). Resolvin D, a pro-resolving lipid mediator, was reported to reduce synovial thickening by modifying macrophages from a high to a low MHC II phenotype and reducing the number of macrophages in the synovium (54). Moreover, upon analyzing single-cell data, FCER1G, HLA-DMB, and HLA-DPA1 were primarily highly expressed in DCs and macrophages in our study. The study conducted by Pinto et al. also revealed that the cells expressing MHC class II genes were macrophages and DCs (55). In the cell-cell interaction network, we found that HLA-DMB and HLA-DPA1 interacted as ligands with the CD4 receptor through the MHC-II signaling pathway. MHC II-like peptide complexes in lipid microdomains of dendritic cells induced the synthesis of IL-12, which initiated the CD4 Th1 phenotype (56). To date, no experiments have been performed to investigate the specific mechanisms of 3 signature genes in the OA synovium, suggesting that the 3 signature genes may be potential therapeutic targets for future OA research.

Genetic variants associated with biomarkers in the MR study can be used as instrumental variables to infer causal relationships between biomarkers and disease (57). Therefore, we conducted in-depth analyses of the causal associations between biomarkers and OA through the MR study. Using eQTL and GWAS data, the main purpose of the two-sample MR was to test whether eQTL variants as instrumental variables mediate their effects on disease by affecting gene expression (58). Previous studies have used the MR method to identify valuable therapeutic targets for OA. For example, based on a MR study, activity-reduced ADAMTS5 was identified as a therapeutic target to reduce the risk of OA (59). In this study, the IVW results showed a significant association between HLA-DMB and the risk of developing OA, and nominally significant associations between FCER1G and HLA-DPA1 and the risk of developing OA. Compared to the IVW results, the MR-Egger results with lower statistical power were not significant. Despite the heterogeneity in the causal estimate of HLA-DPA1 on OA (P heterogeneity < 0.001), the causal effect estimated using the random-effects IVW method remained significant in the absence of horizontal pleiotropy, which might balance the pooled heterogeneity. In addition, the causal effect of the MR study of HLA-DPA1 was supported by the consistent results of WM and IVW. Thus, we observed that the expression of FCER1G, HLA-DMB and HLA-DPA1 was positively associated with OA by a two-sample MR study, demonstrating the therapeutic potential of the 3 biomarkers for OA.

Our study comprehensively elucidated the immune microenvironment of OA. However, there are some limitations in our study, and although more advanced sequencing data were used, the sample size obtained was not large enough, and further expansion of the sample size is needed. Due to the lack of synovial tissue-specific eQTLs, we chose to use whole blood and fibroblast eQTLs as a feasible alternative to explore the potential causal relationships between the expression of the three biomarkers and OA. Although these eQTLs may not perfectly represent eQTLs in synovial tissue, they still provide a valuable avenue for investigating the potential associations between biomarkers and OA in the absence of synovial tissue-specific eQTL data. Furthermore, although the 3 signature genes have been validated by two independent validation datasets, experiments are still needed to verify their mechanisms of action in further studies.

In summary, we identified three promising biomarkers of OA (FCER1G, HLA-DMB, and HLA-DPA1) using multi-omics data combined with bioinformatics and MR approaches. The MR study using large-scale GWAS summary data demonstrated that three biomarkers had causal effects on OA. These findings can provide guidance for future work in uncovering the molecular mechanisms responsible for OA.

## Data availability statement

The datasets presented in this study can be available in the Gene Expression Omnibus database (Accession number: “GSE89408”, “GSE143514”, “GSE55235”, “GSE46750”, and “GSE152805”). Immune-related genes can be obtained from the ImmPort database.

## Author contributions

W-WP: Conceptualization, Methodology, Visualization, Writing – original draft, Writing – review & editing. Y-SC: Methodology, Writing – review & editing. CC: Methodology, Writing – review & editing. F-RZ: Software, Writing – review & editing. QZ: Software, Writing – review & editing. D-YL: Software, Writing – review & editing. NW: Software, Writing – review & editing. X-CQ: Supervision, Writing – review & editing. X-DC: Supervision, Writing – review & editing. H-WD: Conceptualization, Supervision, Writing – review & editing. L-JT: Conceptualization, Supervision, Writing – review & editing.
