# Supplementary material for: Mendelian randomization and transcriptome analysis identified immune-related biomarkers for osteoarthritis
Source: Front Immunol. 2024 Apr 12;15:1334479. doi: 10.3389/fimmu.2024.1334479 (PMC11045931; doi:10.3389/fimmu.2024.1334479)
Supplement: Supplementary file 1 [file DataSheet_1.pdf]

Supplementary Figure 1

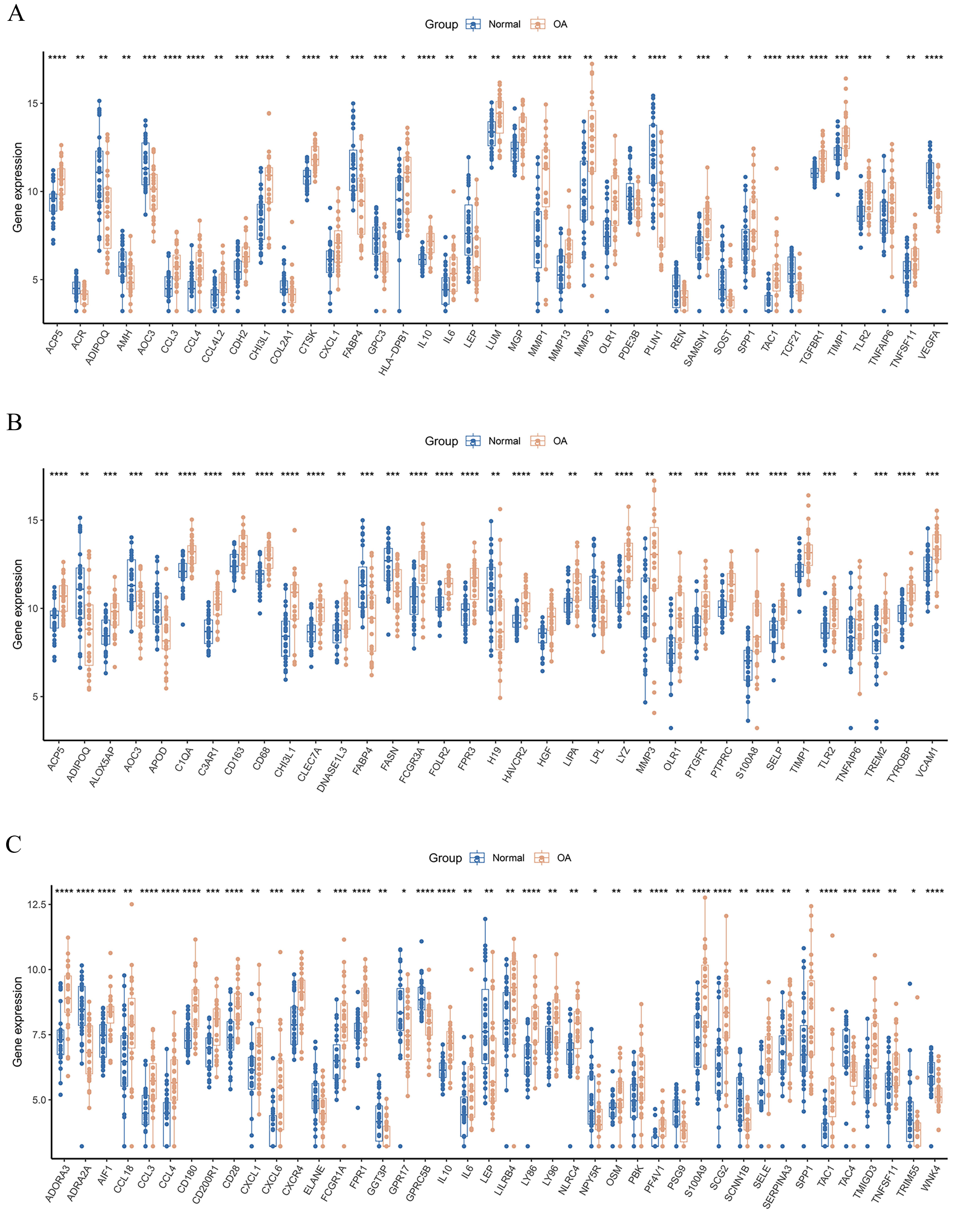

Supplementary Figure 1 Validation of differential expression of OA related genes and inflammatory genes. A Boxplots displaying differentially expressed OA related genes. (B, C) Boxplots displaying differentially expressed OA genes and inflammatory genes. Normal (n = 31), OA (n = 27). The Wilcoxon test in A, B, and C. (*$p < 0.05$, **$p < 0.01$, ***$p < 0.001$, ****$p < 0.0001$).

Supplementary Figure 2

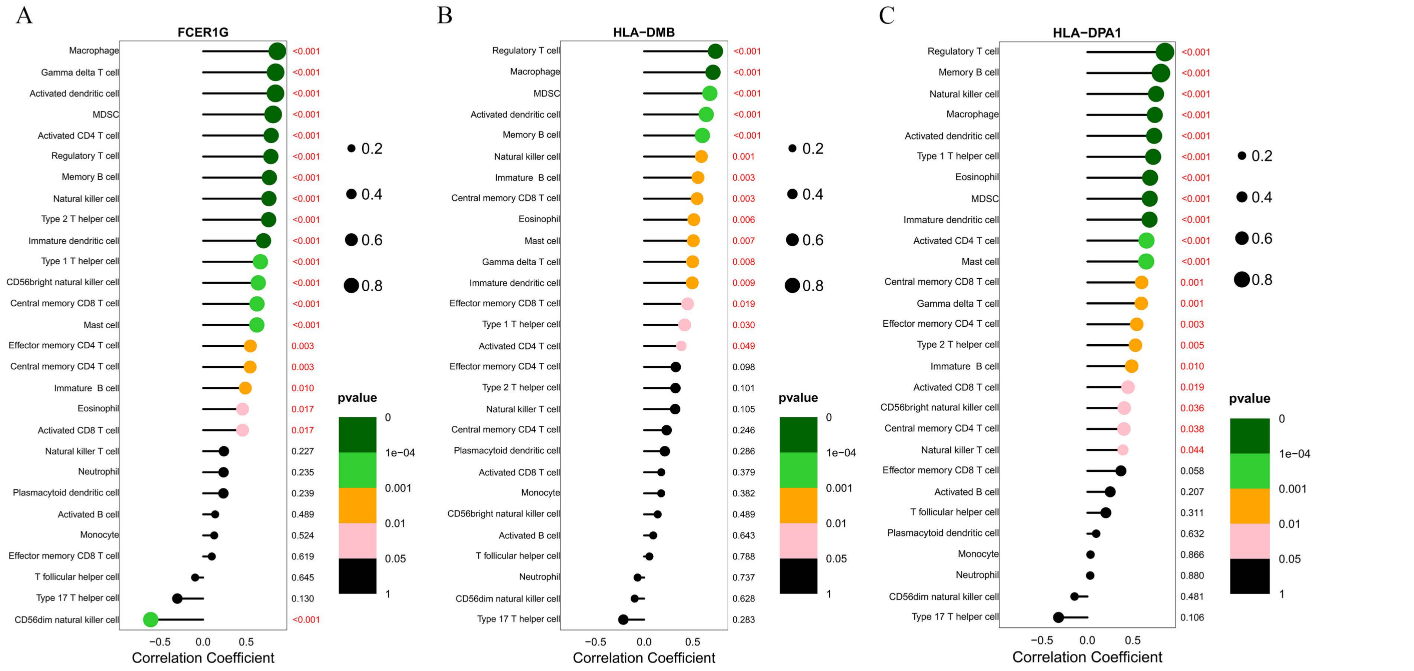

Supplementary Figure 2 Correlation analysis between the levels of infiltration of immune cells and the 3 signature genes. FCER1G (A), HLA-DMB (B), and HLA-DPA1 (C).

Supplementary Figure 3

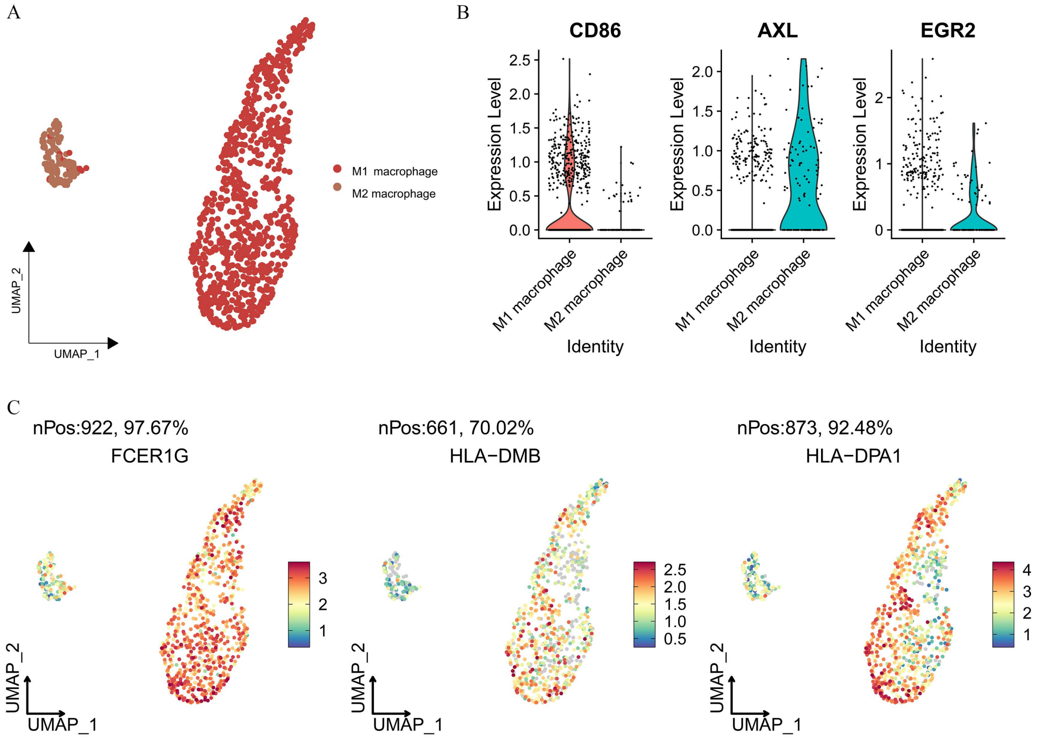

Supplementary Figure 3 Expression of signature genes in M1 macrophages and M2 macrophages. A UMAP plot of the macrophage cluster. B Violin plots showing the expression of marker genes in two macrophage subtypes. C Umap plots showing the expression of signature genes in two macrophage subtypes.

Supplementary Figure 4

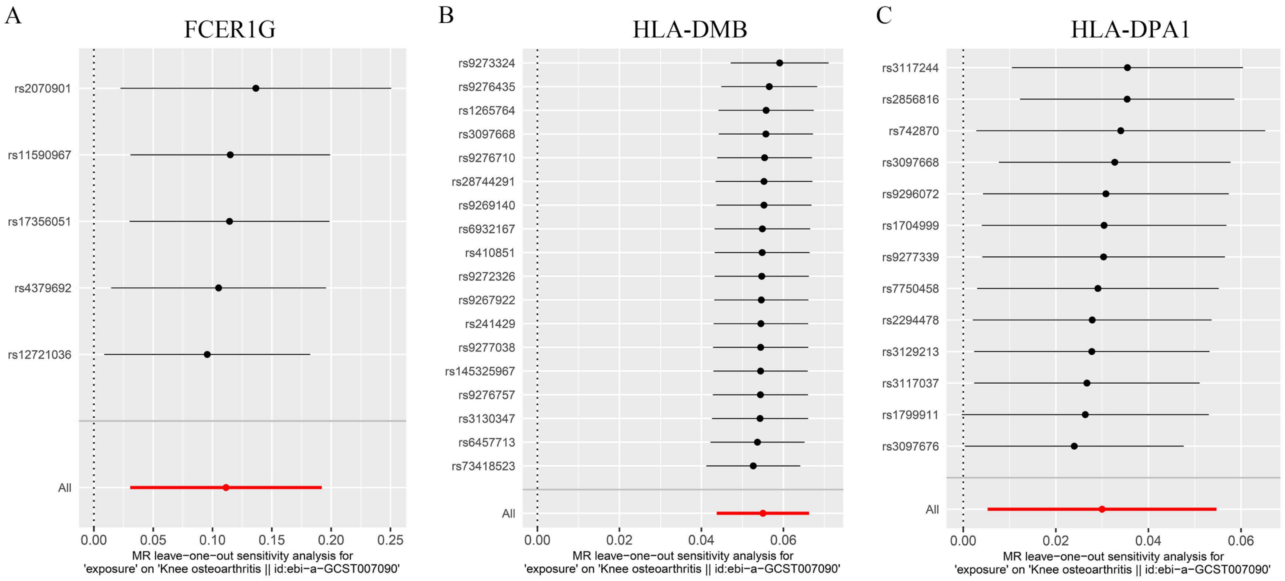

Supplementary Figure 4 Leave-One-Out Plots for biomarkers on OA. FCER1G (A), HLA-DMB (B), and HLA-DPA1 (C).
